# Integrating Feature Selection, Machine Learning, and SHAP Explainability to Predict Severe Acute Pancreatitis

**DOI:** 10.3390/diagnostics15192473

**Published:** 2025-09-27

**Authors:** İzzet Ustaalioğlu, Rohat Ak

**Affiliations:** 1 Department of Emergency Medicine, Gönen State Hospital, 10900 Balıkesir, Türkiye; 2 Department of Emergency Medicine, Health Sciences University, Kartal Dr. Lütfi Kırdar City Hospital, 34865 İstanbul, Türkiye; rohatakmd@gmail.com

**Keywords:** pancreatitis, machine learning, risk assessment, predictive modeling, feature selection

## Abstract

**Background/Objectives**: Severe acute pancreatitis (SAP) carries substantial morbidity and resource burden, and early risk stratification remains challenging with conventional scores that require serial observations. The aim of this study was to develop and compare supervised machine-learning (ML) pipelines—integrating feature selection and SHAP-based explainability—for early prediction of SAP at emergency department (ED) presentation. **Methods**: This retrospective, single-center cohort was conducted in a tertiary-care ED between 1 January 2022 and 1 January 2025. Adult patients with acute pancreatitis were identified from electronic records; SAP was classified per the Revised Atlanta criteria (persistent organ failure ≥ 48 h). Six feature-selection methods (univariate AUROC filter, RFE, mRMR, LASSO, elastic net, Boruta) were paired with six classifiers (kNN, elastic-net logistic regression, MARS, random forest, SVM-RBF, XGBoost) to yield 36 pipelines. Discrimination, calibration, and error metrics were estimated with bootstrapping; SHAP was used for model interpretability. **Results**: Of 743 patients (non-SAP 676; SAP 67), SAP prevalence was 9.0%. Compared with non-SAP, SAP patients more often had hypertension (38.8% vs. 27.1%) and malignancy (19.4% vs. 7.2%); they presented with lower GCS, higher heart and respiratory rates, lower systolic blood pressure, and more frequent peripancreatic fluid (31.3% vs. 16.9%) and pleural effusion (43.3% vs. 17.5%). Albumin was lower by 4.18 g/L, with broader renal–electrolyte and inflammatory derangements. Across the best-performing models, AUROC spanned 0.750–0.826; the top pipeline (RFE–RF features + kNN) reached 0.826, while random-forest-based pipelines showed favorable calibration. SHAP confirmed clinically plausible contributions from routinely available variables. **Conclusions**: In this study, integrating feature selection with ML produced accurate and interpretable early prediction of SAP using data available at ED arrival. The approach highlights actionable predictors and may support earlier triage and resource allocation; external validation is warranted.

## 1. Introduction

Severe acute pancreatitis (SAP) is a life-threatening form of acute pancreatitis (AP) that imposes a significant clinical and economic burden [1]. Globally, AP is one of the most common gastrointestinal emergencies, with an incidence of approximately 34 per 100,000 population [2,3]. While the majority of AP cases are mild and self-limited, about 20% progress to SAP, often accompanied by pancreatic necrosis and multi-organ failure, with reported mortality rates as high as 20–40% [4]. Patients with SAP frequently require intensive care support, and outcomes remain poor even with aggressive management—a recent Australasian ICU cohort reported an in-hospital mortality of 11.6% among AP patients admitted to intensive care [5]. These observations emphasize the urgent need for early identification of high-risk patients to facilitate prompt interventions and improve survival.

Machine learning (ML) has emerged as a promising approach for developing predictive models in clinical medicine [6]. Traditional scoring systems for pancreatitis severity (e.g., Ranson criteria, APACHE II, BISAP) are valuable but rely on a fixed set of clinical variables and often require 24–48 h of observation, which can delay critical decision-making [7]. In contrast, ML algorithms automatically capture complex nonlinear relationships in large datasets, enabling more accurate and earlier predictions of adverse outcomes [8]. Recent studies have shown that ML models can significantly outperform conventional severity scores in predicting SAP outcomes [9,10]. Equally importantly, advances in explainable artificial intelligence—particularly Shapley Additive Explanations (SHAP)—now allow clinicians to interpret ML model outputs by quantifying each feature’s contribution to a prediction [11]. This interpretability fosters trust and provides insight into key prognostic factors, addressing a common barrier to clinical adoption of AI. Furthermore, ML-based risk stratification can be performed at the time of hospital admission, enabling earlier triage of SAP patients to intensive care or aggressive therapy, which is critical for improving outcomes.

Recent work has increasingly leveraged machine learning (ML) for early severity stratification in acute pancreatitis. Yin et al. used automated ML to screen multiple algorithms for early identification of severe acute pancreatitis (SAP), highlighting the feasibility of data-driven model selection in routine clinical cohorts [12].

Hong et al. developed an interpretable random-forest model to predict SAP, reporting competitive discrimination and emphasizing model transparency [13]. Beyond tabular clinical variables, Kui et al. introduced the EASY-APP tool to flag high-risk patients soon after admission, illustrating pragmatic deployment pathways [14]. Image-based deep learning has also been explored: Liang et al. trained a 3D DenseNet on contrast-enhanced CT for early severity grading and Chen et al. validated deep learning on non-enhanced CT to predict severity [15,16]. Relatedly, Kiss et al. applied an extreme gradient boosting model to predict necrotizing pancreatitis in a large multicenter cohort and demonstrated that machine-learning-based approaches can effectively support early identification of patients at risk of severe disease progression [6]. In contrast to prior studies, the present work adopts a comprehensive and systematic approach by combining six complementary feature-selection strategies with six diverse classifiers, yielding 36 model pipelines evaluated under a single protocol. Beyond discrimination, our analysis includes calibration assessment through Brier scores and reliability plots, with all metrics reported alongside bootstrap-derived 95% confidence intervals to provide robust estimates. Importantly, our models rely solely on clinical, laboratory, and early imaging data available at emergency department presentation, ensuring maximal clinical applicability for early triage. Furthermore, we integrate global and patient-level SHAP analyses to enhance interpretability and clinician trust. By identifying a parsimonious yet predictive set of routinely collected variables, our study supports the development of practical, explainable, and implementable risk-stratification tools for severe acute pancreatitis.

In this study, feature selection, machine learning modeling, and SHAP explainability were integrated to predict the development of SAP in its early phase. Multiple ML models were developed using various feature selection techniques and their performance was compared in identifying patients at risk of severe disease. Additionally, SHAP analysis was applied to the best-performing model to interpret feature importance, with the goal of providing an explainable predictive tool that can assist in early clinical decision-making for acute pancreatitis.

## 2. Materials and Methods

### 2.1. Study Design and Setting

This study was designed as a single-center, retrospective observational cohort conducted in the Emergency Medicine Department of Kartal Dr. Lütfi Kırdar City Hospital (Istanbul, Türkiye). The study period spanned from 1 January 2022 through 1 January 2025, during which all eligible cases were identified and analyzed. No interventions were applied as this was an observational study. The Institutional Ethics Committee approved the study protocol (Decision No: 2025/010.99/17/42; Date: 25 June 2025), and the requirement for individual informed consent was waived due to the retrospective design.

### 2.2. Study Population and Data Collection

Adult patients (≥18 years old) who presented to the emergency department and were diagnosed with acute pancreatitis (AP) during the study period were retrospectively identified from the hospital information system using diagnosis codes and clinical records. AP was defined according to the 2012 Revised Atlanta Classification, requiring at least two of the following: (1) characteristic abdominal pain; (2) serum amylase and/or lipase ≥3 times the upper limit of normal; and (3) imaging findings consistent with AP [4]. Severe AP (SAP) was defined as AP with persistent organ failure lasting more than 48 h. Patients were stratified into SAP and non-SAP groups. Exclusion criteria were as follows: patients with chronic liver disease, chronic renal disease, hematologic disorders, recurrent/chronic/traumatic/idiopathic pancreatitis, pancreatic cancer, or history of pancreatic resection; patients who had undergone chemoradiotherapy; pregnant patients; and patients with incomplete medical records for key variables or unknown outcomes.

Demographic information included age and sex. Comorbidities and medical history comprised hypertension, diabetes, malignancy, and other chronic illnesses, recorded as binary variables for presence or absence. Clinical presentation variables encompassed vital signs at admission (heart rate, blood pressure, respiratory rate, body temperature, and peripheral oxygen saturation), Glasgow Coma Scale (GCS) score, and physical examination findings. Laboratory measurements consisted of initial blood test results, including complete blood count (such as white blood cell count and neutrophil percentage), metabolic panel parameters (electrolytes such as calcium and potassium, renal function tests including blood urea nitrogen and creatinine, and bicarbonate levels), as well as liver function and pancreatic enzymes (albumin, bilirubin, alanine aminotransferase, amylase, lipase, and gamma-glutamyl transferase). Inflammatory markers (C-reactive protein and procalcitonin) and coagulation parameters (prothrombin time, activated partial thromboplastin time, and international normalized ratio) were also recorded. Imaging and early complications were documented from abdominal ultrasound or computed tomography at presentation, including the presence of gallstones, peripancreatic fluid collections, or pleural effusions, along with any organ failure identified at admission.

All data were systematically collected and entered into a research database.

### 2.3. Outcome Definition

The primary outcome was the development of SAP during the hospital stay, as opposed to non-severe disease. SAP was defined in accordance with the Revised Atlanta Classification criteria as acute pancreatitis accompanied by persistent organ failure lasting more than 48 h. Organ failure was assessed in three organ systems (respiratory, cardiovascular, or renal) using the modified Marshall scoring system, with a score ≥2 in any system indicating organ failure. If such organ dysfunction persisted for >48 h despite treatment, the episode was classified as severe [4]. Patients who fulfilled these criteria at any point after presentation were categorized as having developed SAP, whereas those who recovered without persistent organ failure were considered non-severe cases. The classification of each case was made retrospectively by evaluating clinical notes, ICU admissions, and organ support requirements recorded in the patient’s chart.

### 2.4. Feature Selection and Machine Learning Models

Accurate prediction of severe acute pancreatitis requires handling numerous, often correlated clinical and laboratory variables. To address this, six complementary feature selection strategies were implemented that have been widely applied in biomedical prediction tasks. Univariate AUROC filtering prioritizes single predictors with strong discriminatory capacity, whereas recursive feature elimination with random forest (RFE-RF) iteratively removes less informative variables based on model performance [17]. Minimum redundancy–maximum relevance (mRMR) selects features that are both relevant to the outcome and minimally collinear [18]. Regularization-based methods, such as LASSO and elastic net, shrink regression coefficients to stabilize selection when predictors are highly correlated [19]. Finally, the Boruta algorithm identifies all features significantly associated with the outcome using random forest permutations [20]. These approaches are particularly applicable to acute pancreatitis, where diverse routine variables—including inflammatory markers, electrolytes, and imaging findings—may overlap but only a subset contributes meaningfully to disease severity [8,9]. The variables selected by each method are summarized in Supplementary Table S1.

Six machine learning classifiers were evaluated in parallel to represent complementary modeling paradigms. k-nearest neighbors (kNN) was included as a non-parametric comparator sensitive to local data structure. Logistic regression with elastic net penalty provided a regularized linear benchmark. Multivariate adaptive regression splines (MARS) flexibly captured non-linear effects in moderate-sized datasets [21]. Random forest and extreme gradient boosting (XGBoost) represented ensemble tree-based methods capable of modeling complex, high-order feature interactions, which are common in multifactorial diseases such as pancreatitis [22,23]. A support vector machine with a radial basis function kernel (SVM-RBF) was included for its robustness in handling non-linear boundaries in high-dimensional data [24]. This framework yielded 36 pipelines (6 × 6 combinations), enabling systematic evaluation of feature–model pairings to identify the most effective strategies for early risk stratification in acute pancreatitis.

### 2.5. Statistical Analysis

All statistical analyses were performed using R version 4.4.2 (R Foundation for Statistical Computing, Vienna, Austria). Continuous variables were first assessed for distributional normality using histograms in conjunction with the Kolmogorov–Smirnov test. Normally distributed variables were presented as mean ± standard deviation (SD) and compared using the independent samples *t*-test, with mean difference (95% confidence interval [CI]) additionally reported when *p* < 0.05. Non-normally distributed variables were expressed as median [interquartile range, IQR] and compared using the Mann–Whitney U test. Categorical variables were summarized as counts (*n* [%]) and compared with the χ^2^ test or Fisher’s exact test where appropriate. For machine-learning model development, the dataset was randomly split into a training set (70%) and an independent test set (30%). Within the training set, 5-fold cross-validation was performed to tune hyperparameters and to evaluate feature selection and classification pipelines. Six complementary feature selection strategies were applied—univariate AUC filter, recursive feature elimination (RFE), minimum redundancy–maximum relevance (mRMR), least absolute shrinkage and selection operator (LASSO), elastic net regularization, and Boruta—each combined with six classifiers: k-nearest neighbors (kNN), logistic regression with elastic net penalty, multivariate adaptive regression splines (MARS), random forest, support vector machine with radial basis kernel (SVM-RBF), and extreme gradient boosting (XGBoost). Model performance was quantified using area under the receiver operating characteristic curve (AUROC), F1 score, precision, recall, log loss, and Brier score. Final performance metrics were reported on the independent 30% test set, with 95% CIs obtained via bootstrapping (*n* = 1000 resamples). Pairwise AUROC comparisons were conducted using DeLong’s test with false discovery rate (FDR) correction for multiple comparisons. Calibration was assessed using Brier score and calibration plots. Model explainability was examined with Shapley additive explanations (SHAP). Mean absolute SHAP values were used to rank predictors, and individual SHAP plots illustrated the contribution of each variable to patient-level predictions.

### 2.6. Proposed Approach

A graphical abstract summarizing the end-to-end pipeline is provided (Graphical Abstract). The approach proceeds in the following steps: (i) retrospective cohort identification (1 January 2022–1 January 2025) with eligibility per Revised Atlanta and prespecified exclusions; (ii) extraction of ED-arrival variables (demographics, comorbidities, vital signs, Glasgow Coma Scale, routine laboratories, and early imaging flags); (iii) outcome assignment as severe acute pancreatitis (persistent organ failure ≥ 48 h, modified Marshall ≥ 2); (iv) random partitioning into training (70%) and independent test (30%) sets with five-fold cross-validation within the training set; (v) feature selection using six strategies (univariate AUC, RFE-RF, mRMR, LASSO, elastic net, Boruta); (vi) model fitting with six classifiers (kNN, elastic-net logistic regression, MARS, random forest, SVM-RBF, XGBoost); (vii) evaluation using AUROC, F1, precision, recall, log loss, and Brier score, with pairwise AUROC compared by DeLong test and calibration inspected by Brier and plots; (viii) explainability with SHAP at global and patient levels; and (ix) selection of the best-performing pipeline as the candidate ED risk-scoring tool.

## 3. Results

All analyses were executed on a 2024 MacBook Air 15″ (Apple M3 system-on-chip: 8-core CPU [4 performance/4 efficiency], 10-core GPU, 16-core Neural Engine; 8 GB unified memory). Computations were performed in R 4.4.2 within RStudio (2024.12.0 Build 467) (Posit, macOS) using commonly available libraries for modeling and evaluation (e.g., glmnet, ranger, xgboost, e1071, earth, Boruta, mRMRe, pROC, and SHAP utilities such as fastshap/shapviz). GPU acceleration was not required; model training and evaluation were conducted on CPU. SI units were used throughout: electrolytes and metabolites in mmol/L (unless otherwise specified), albumin in g/L, enzymes in U/L, bilirubin in μmol/L, temperature in °C, blood pressure in mmHg, heart rate in beats/min, respiratory rate in breaths/min, oxygen saturation as %, and time in hours. For binary classification with SAP as the positive class, metrics on the independent 30% test set were defined and reported with 95% confidence intervals from bootstrap resampling (*n* = 1000) as follows:

Precision = TP/(TP + FP);

Recall (Sensitivity) = TP/(TP + FN);

Specificity = TN/(TN + FP);

F1 = 2 × (Precision × Recall)/(Precision + Recall);

Accuracy = (TP + TN)/(TP + TN + FP + FN);

Log loss = −(1/N) Σ[y·ln(p) + (1 − y)·ln(1 − p)];

Brier score = (1/N) Σ(p − y)^2^.

AUROC summarized discrimination as the probability that a randomly chosen SAP case receives a higher predicted risk than a non-SAP case; threshold-dependent metrics (precision, recall, F1, specificity, accuracy) were reported at a probability threshold chosen to maximize F1 on the training set, and calibration was examined using the Brier score and reliability plots.

A total of 743 patients were included (non-severe acute pancreatitis [non-SAP], *n* = 676; severe acute pancreatitis [SAP], *n* = 67). Baseline characteristics and clinical findings are summarized in Table 1. There was no statistically significant difference between groups in age (49 ± 17 vs. 49 ± 19 years; *p* = 0.980) or sex (50.6% vs. 59.7% male; *p* = 0.155). Among comorbidities, hypertension was more frequent in the SAP group (27.1% vs. 38.8%; *p* = 0.042), and malignancy was also more common in SAP (7.2% vs. 19.4%; *p* < 0.001); other comorbidities showed no statistically significant difference. Biliary etiology did not differ between groups (41.3% vs. 31.3%; *p* = 0.114). The median Glasgow Coma Scale (GCS) was lower in SAP (15.0 [14.0–15.0] vs. 15.0 [15.0–15.0]; *p* < 0.001). Vital signs favored higher acuity in SAP: heart rate was higher by Δ 7.45 beats/min (95% CI 2.41 to 12.48; *p* = 0.004), respiratory rate by Δ 2.68 breaths/min (95% CI 1.22 to 4.13; *p* < 0.001), and systolic blood pressure was lower by Δ 5.10 mmHg (95% CI 0.75 to 9.45; *p* = 0.022); temperature was higher by Δ 0.17 °C (95% CI 0.02 to 0.31; *p* = 0.029). Oxygen saturation (SpO_2_) and Shock Index were worse in SAP (both *p* ≤ 0.018). On imaging/clinical assessment, peripancreatic fluid (16.9% vs. 31.3%; *p* = 0.003) and pleural effusion (17.5% vs. 43.3%; *p* < 0.001) were more frequent in SAP (Table 1).

Laboratory findings are shown in Table 2. Protein and hepatobiliary markers indicated more systemic involvement in SAP: albumin was lower by Δ 4.18 g/L (95% CI 2.63 to 5.72; *p* < 0.001), and median direct bilirubin and gamma-glutamyl transferase were higher (both *p* ≤ 0.002). Renal/electrolyte and acid–base parameters also differed: blood urea nitrogen and creatinine were higher (both *p* ≤ 0.001), calcium was lower (*p* < 0.001), potassium was higher by Δ 0.15 mmol/L (95% CI 0.04 to 0.27; *p* = 0.009), and bicarbonate was lower by Δ 1.10 mmol/L (95% CI 0.23 to 1.96; *p* = 0.014). Inflammation/hematology and coagulation profiles showed higher white blood cell count and neutrophils (both *p* ≤ 0.023) and a longer prothrombin time by Δ 0.59 s (95% CI 0.03 to 1.15; *p* = 0.040). Other markers, including alanine aminotransferase, lipase, C-reactive protein, procalcitonin, lactate dehydrogenase, activated partial thromboplastin time, and international normalized ratio, showed no statistically significant difference (all *p* ≥ 0.060).

**Figure 1:**
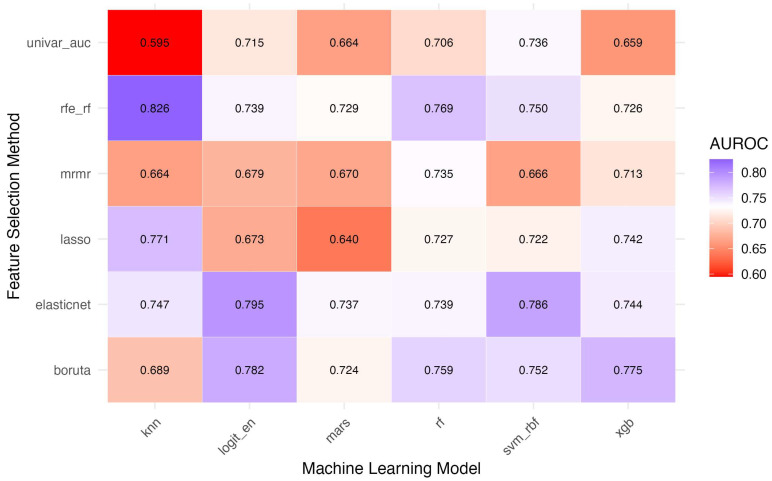
Heatmap of the area under the receiver operating characteristic curve (AUROC) values for different feature selection (FS) methods and machine learning (ML) models in predicting severe acute pancreatitis. *Feature selection methods*: univar_auc = Univariate AUC-based Selection; rfe_rf = Recursive Feature Elimination with Random Forest; mrmr = Minimum Redundancy Maximum Relevance; lasso = Least Absolute Shrinkage and Selection Operator; elasticnet = Elastic Net Regularization; boruta = Boruta Algorithm. *Machine learning models*: knn = k-Nearest Neighbors; logit_en = Logistic Regression with Elastic Net Penalty; mars = Multivariate Adaptive Regression Splines; rf = Random Forest; svm_rbf = Support Vector Machine with Radial Basis Function Kernel; xgb = Extreme Gradient Boosting.

**Figure 2:**
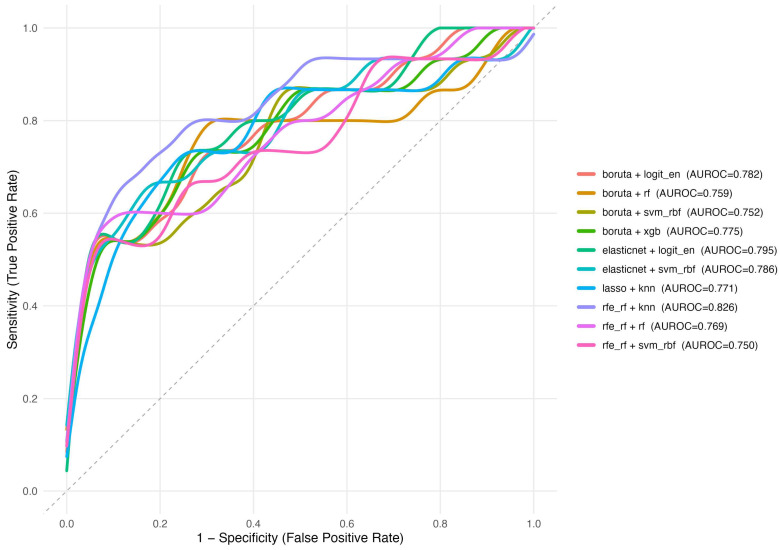
Receiver Operating Characteristic (ROC) Curves of the Top 10 Model Combinations. AUROC = area under the receiver operating characteristic curve; *Feature selection methods*: univar_auc = Univariate AUC-based Selection; rfe_rf = Recursive Feature Elimination with Random Forest; mrmr = Minimum Redundancy Maximum Relevance; lasso = Least Absolute Shrinkage and Selection Operator; elasticnet = Elastic Net Regularization; boruta = Boruta Algorithm. *Machine learning models*: knn = k-Nearest Neighbors; logit_en = Logistic Regression with Elastic Net Penalty; mars = Multivariate Adaptive Regression Splines; rf = Random Forest; svm_rbf = Support Vector Machine with Radial Basis Function Kernel; xgb = Extreme Gradient Boosting. The dashed diagonal line represents the line of no discrimination (AUROC = 0.5).

Model performance across 36 feature selection–model combinations is detailed in Table 3 and visualized in Figure 1 (heatmap) and Figure 2 (receiver operating characteristic curves). Among the top 10 performing models, area under the receiver operating characteristic curve (AUROC) values ranged from 0.750 to 0.826. The highest AUROC was observed for recursive feature elimination with random-forest features combined with k-nearest neighbors (RFE-RF + kNN; AUROC 0.826, 95% CI 0.686–0.965), followed by elastic-net-selected support vector machine (0.786, 95% CI 0.637–0.936) and elastic-net logistic regression (0.795, 95% CI 0.661–0.929). Boruta + XGBoost achieved AUROC 0.775 (95% CI 0.628–0.921). Pairwise DeLong comparisons showed small absolute ΔAUROC values (–0.13 to +0.17) with no statistically significant differences after false discovery rate correction (all q ≥ 0.97), indicating that RFE-RF + kNN, while numerically highest, was statistically comparable to other leading models.

Discrimination–calibration trade-offs were observed. For example, some models combined moderate discrimination with favorable calibration metrics (e.g., minimum redundancy–maximum relevance [mRMR] + random forest with the lowest Brier score of 0.065 and low log loss), whereas the top-AUROC model (RFE-RF + kNN) exhibited higher recall (0.733) at the expense of precision (0.204), reflected in a higher log loss (0.504) and Brier score (0.159) (Table 3). Overall, random-forest-based pipelines tended to offer robust calibration, while kNN and support vector machine emphasized sensitivity/recall among the best performers.

**Figure 3:**
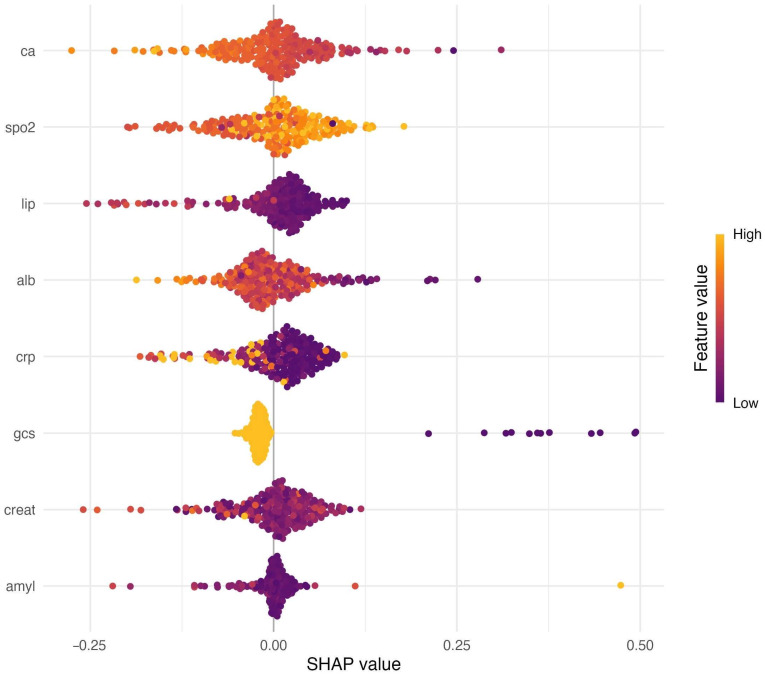
SHAP summary plot for the best-performing model (RFE-RF + kNN). Each dot represents one patient. The horizontal position indicates the SHAP value (feature contribution to predicted probability), and color encodes the feature value (purple = low, yellow = high). Features are ordered by their mean absolute SHAP importance. Abbreviations: Alb, albumin; CRP, C-reactive protein; Ca, calcium; GCS, Glasgow Coma Scale; SpO_2_, peripheral oxygen saturation; Creat, creatinine; Lip, lipase; Amyl, amylase.

Model explainability with SHAP (Shapley additive explanations) for the best-performing pipeline (RFE-RF + kNN) highlighted clinically plausible contributions from routinely available variables, with both higher-risk and protective directions represented across the feature spectrum (Figure 3). Features are displayed in descending mean absolute SHAP importance, with color encoding raw values to aid clinical interpretation.

## 4. Discussion

In this study, multiple feature selection strategies were applied in combination with diverse machine learning models to predict SAP. The models demonstrated a consistent ability to distinguish patients who later developed severe disease from those with non-severe presentations. Importantly, pipelines that reduced the number of predictors still preserved their predictive capacity, suggesting that a focused set of routinely available clinical and laboratory variables may be sufficient for effective risk stratification. In addition, SHAP-based analysis of the best-performing model highlighted clinically meaningful contributors to severe outcomes, including markers of systemic inflammation, metabolic disturbance, and physiological instability. These results indicate that integrating feature selection with machine learning provides a practical and interpretable framework for early identification of patients at risk of SAP.

Early recognition of SAP is clinically crucial because SAP carries a high risk of organ failure and death. SAP is associated with mortality rates up to 20–30%, especially when persistent organ failure or infected necrosis develops [8,11]. Timely identification of patients likely to deteriorate allows prompt intensive management (aggressive fluid resuscitation, organ support, and specialist care), measures which have been shown to improve outcomes [25]. Traditional severity scoring systems (e.g., Ranson criteria, BISAP) aid initial risk stratification, but they have important limitations in the early phase. Ranson’s score requires 48 h of data and thus delays risk stratification, and while BISAP can be calculated at admission, its sensitivity for severe disease is suboptimal despite good specificity [26]. In this cohort, some patients who eventually developed SAP may not have been flagged by these scores at baseline. This gap highlights the need for more agile predictive methods. By providing an accurate risk assessment soon after admission, ML-based models developed in this study can facilitate triage decisions for high-risk patients, potentially before clinical deterioration becomes apparent. Early SAP recognition is therefore not only prognostically important but also essential for guiding timely interventions that may mitigate disease severity.

Our findings are in line with emerging literature that applies ML to AP. A growing number of studies report that ML models outperform conventional scoring systems in predicting AP severity and outcomes [27]. Traditional scores offer a useful baseline, but they often lack precision in the early stage of pancreatitis [23]. For example, Ranson and BISAP scores, while widely used, do not reliably identify all high-risk patients at admission [28]. In a recent systematic review, the pooled prognostic accuracy of Ranson and BISAP was moderate, and no single scoring system was clearly superior for predicting SAP or mortality [27]. In contrast, ML techniques can automatically detect complex, non-linear interactions among variables that traditional methods might miss. In this study, the ML model likely capitalized on subtler patterns in the data (e.g., combinations of lab trends and vital signs) to improve predictive performance. This advantage of ML has been demonstrated by others: Thapa et al. developed an XGBoost model on a large electronic health record dataset (>60,000 AP cases) and achieved an AUROC of 0.92 for early prediction of SAP, significantly higher than the AUROCs of bedside scores like HAPS and BISAP [8]. Similarly, an artificial neural network model described by Ding et al. outperformed logistic regression, Ranson, and even ICU scores like SOFA in predicting in-hospital mortality for AP [29]. López Gordo et al. reported that an ML model (XGBoost) attained an AUC of 0.93 in forecasting SAP, substantially exceeding the accuracy of admission BISAP (AUC ~0.74) and APACHE II (AUC ~0.81) [23]. These comparative gains underscore that ML-based approaches can provide more sensitive and specific early warning of severe disease than legacy scoring systems. In practical terms, this means fewer false negatives and potentially more lead time to intervene before fulminant SAP ensues.

Beyond raw performance, a key advantage of the proposed approach is the interpretability added by SHAP analysis. One common criticism of ML models in medicine is their “black box” nature, which can limit clinician trust and hinder adoption. By using SHAP values, we addressed this concern—the model not only predicts risk but also explains why a given patient is at high risk. The SHAP summaries highlighted clinically intuitive predictors. This interpretability is invaluable: it provides reassurance that the model is relying on meaningful patterns rather than spurious correlations, and it allows clinicians to see how patient-specific factors contribute to the risk score. Recent studies have similarly incorporated explainable AI in pancreatitis care. Li et al. built an ML model for 30-day mortality in SAP and used SHAP to identify important predictors, finding that use of vasopressors, Charlson index, hypoxemia, and hyperglycemia were among the strongest contributors to mortality risk [30]. In another study on infected pancreatic necrosis, an explainable random forest model revealed that clinical indicators like persistent organ failure, high APACHE II, and elevated BISAP score were the top drivers of mortality predictions [31]. These examples mirror the findings of this study—many of the variables flagged by SHAP analysis are well-known risk factors. This convergence is encouraging, as it indicates ML models are capturing real pathophysiologic risk signals. Explainable outputs also enable clinicians to verify that a model’s reasoning aligns with medical knowledge; for instance, if a model predicts a patient will develop SAP primarily due to a rising blood urea nitrogen and tachycardia, a physician can understand and potentially act on those specific findings [32]. In essence, SHAP transforms an otherwise complex ensemble model into a form of decision support that is transparent and easier to integrate into clinical reasoning. We expect that this clarity will improve user acceptance of ML tools. It may also uncover new insights—for example, if the model had identified an unexpected variable as highly influential, that could prompt further investigation into its role in SAP, thereby generating new hypotheses for research.

The integration of ML prediction and explainability holds promise to enhance acute pancreatitis management. An accurate early warning model for SAP could be deployed as a decision-support tool in emergency departments or hospital wards. For instance, a web-based calculator or an electronic health record alert could use the patient’s initial data (labs, vitals, etc.) to compute a risk score for severe pancreatitis [31]. High-risk patients could then be prioritized for intensive monitoring, aggressive fluid management, nutritional support, and early specialist involvement, even before organ failure manifests. This proactive approach aligns with the current trend of personalized medicine—tailoring the level of care to the individual’s risk profile. From a systems perspective, better early risk stratification could optimize resource allocation, ensuring that ICU beds and interventions are reserved for those most likely to benefit [23]. The simplicity of the model (after feature selection) also indicates that it could be relatively easy to implement; it uses routinely available variables, making it feasible for diverse hospitals including those without advanced tests.

Although our study did not specifically evaluate downstream clinical outcomes such as length of ICU or hospital stay, early risk stratification has been shown to improve triage and enable timely interventions in SAP, which may shorten ICU admission and overall hospitalization [25,27]. Previous studies have reported that early recognition of high-risk patients facilitates prompt fluid resuscitation, nutritional support, and specialist consultation, potentially reducing complications and mortality [8,23]. Integrating our ML-based prediction model into clinical workflows could therefore not only improve risk stratification but also contribute to optimizing resource utilization and patient outcomes. Prospective multicenter studies are warranted to confirm these potential benefits and quantify their effect on prognosis.

### Limitations

This study has some limitations that should be considered when interpreting the results. Its retrospective and single-center design may limit external validity, as patient populations and management practices can vary across institutions. Although the dataset included a broad range of routinely collected clinical, laboratory, and imaging parameters, certain potentially relevant variables such as advanced imaging scores or novel biomarkers were not available. Moreover, the absence of external validation raises the possibility that the performance observed in this cohort may not be fully reproducible in different healthcare settings. Finally, while SHAP provided insight into feature contributions, interpretability remains dependent on the quality and completeness of the input data. These factors should be considered when interpreting the results, and future multicenter, prospective studies are needed to confirm the clinical applicability of the proposed models.

## 5. Conclusions

This study shows that an integrated framework combining complementary feature selection techniques with diverse machine-learning models can support early risk stratification for severe acute pancreatitis using information available at emergency department presentation. The approach yields clinically coherent explanations through SHAP, linking predictions to routinely collected variables in a way that is transparent and operationally meaningful for frontline decision-making. In contrast to prior reports that focus on single algorithms or narrow sets of predictors, our work implements a systematically benchmarked, end-to-end pipeline in which multiple selection strategies are paired with heterogeneous classifiers under a single protocol. The evaluation explicitly addresses calibration and estimates uncertainty for all key metrics, rather than emphasizing discrimination alone. By restricting inputs to admission-time clinical, laboratory, and early imaging variables, the framework targets the phase when triage decisions must be made, and it couples this with unified, global and patient-level explainability to enhance clinical credibility and potential adoption. Together, these elements provide a practical template for building early, interpretable, and deployment-oriented tools for pancreatitis severity assessment. The proposed framework can be integrated into clinical workflows as a decision-support layer to prioritize monitoring, guide timely escalation of care, and optimize resource allocation. Future work should include external, multicenter validation and prospective implementation studies to quantify its effect on downstream outcomes and care processes. Evaluations that examine clinical impact (including length of ICU and hospital stay, complication profiles, and resource use) will be essential to translate methodological advances into measurable patient benefit.

## Figures and Tables

**Table 1:** Baseline Characteristics, Vital Signs, and Early Imaging at Emergency Department Presentation.

Variable	Non-SAP (*n* = 676)	SAP (*n* = 67)	*p*	Mean Difference (95% CI)
Demographics				
Age (years)	49 ± 17	49 ± 19	0.980	
Male sex (*n* [%])	342 (50.6%)	40 (59.7%)	0.155	
Comorbidities				
Coronary artery disease	55 (8.1%)	10 (14.9%)	0.061	
Chronic obstructive pulmonary disease	34 (5.0%)	1 (1.5%)	0.357	
Diabetes mellitus	113 (16.7%)	14 (20.9%)	0.386	
Hypertension	183 (27.1%)	26 (38.8%)	0.042 *	
Malignancy	49 (7.2%)	13 (19.4%)	<0.001 *	
Etiology				
Biliary etiology	279 (41.3%)	21 (31.3%)	0.114	
Neurological/Score				
Glasgow Coma Scale (median [IQR])	15.0 [15.0–15.0]	15.0 [14.0–15.0]	<0.001 *	
Vital Signs				
Heart rate (beats/min)	85 ± 15	93 ± 20	0.004 *	Δ −7.45 (95% CI −12.48 to −2.41)
Respiratory rate (breaths/min)	18 ± 4	21 ± 6	<0.001 *	Δ −2.68 (95% CI −4.13 to −1.22)
Systolic blood pressure (mmHg)	120 ± 15	115 ± 17	0.022 *	Δ 5.10 (95% CI 0.75 to 9.45)
Diastolic blood pressure (mmHg)	75 ± 10	72 ± 14	0.148	
Oxygen saturation (%) (median [IQR])	97 [95–98]	96 [94–98]	0.018 *	
Temperature (°C)	36.8 ± 0.5	37.0 ± 0.6	0.029 *	Δ −0.17 (95% CI −0.31 to −0.02)
Shock Index (median [IQR])	0.7 [0.6–0.8]	0.8 [0.7–1.0]	<0.001 *	
Clinical/Imaging Findings				
Peripancreatic fluid (*n* [%])	114 (16.9%)	21 (31.3%)	0.003 *	
Pleural effusion (*n* [%])	118 (17.5%)	29 (43.3%)	<0.001 *	

SAP, severe acute pancreatitis; IQR, interquartile range; CI, confidence interval. * indicates statistical significance at *p* < 0.05.

**Table 2:** Laboratory Findings at Emergency Department Presentation.

Variable	Non-SAP (*n* = 676)	SAP (*n* = 67)	*p*	Mean Difference (95% CI)
Proteins/Enzymes & Hepatobiliary				
Albumin (g/L)	37.8 ± 4.9	33.6 ± 6.2	<0.001 *	Δ 4.18 (95% CI 2.63 to 5.72)
Alkaline phosphatase (U/L)	76.0 [52.0–104.0]	91.0 [55.5–131.0]	0.033 *	
Alanine aminotransferase (U/L)	27.0 [9.0–82.0]	36.0 [14.5–111.0]	0.171	
Aspartate aminotransferase (U/L)	26.0 [15.0–51.0]	33.0 [16.5–73.0]	0.048 *	
Gamma-glutamyl transferase (U/L)	38.0 [25.0–52.0]	52.0 [32.0–74.0]	<0.001 *	
Total bilirubin (µmol/L)	17.4 [11.7–27.9]	16.5 [11.4–23.5]	0.249	
Direct bilirubin (µmol/L)	6.0 [4.3–8.6]	7.0 [5.4–9.9]	0.002 *	
Pancreatic Enzymes				
Amylase (U/L])	576.0 [347.2–1008.2]	648.0 [365.5–1248.5]	0.172	
Lipase (U/L)	635.0 [352.5–1103.0]	655.0 [362.0–1387.0]	0.438	
Renal/Electrolytes & Acid–Base				
Blood urea nitrogen (mmol/L)	4.8 [3.7–6.4]	5.6 [4.2–8.3]	0.001 *	
Creatinine (µmol/L)	60.5 [50.1–75.6]	74.7 [56.0–84.8]	<0.001 *	
Sodium (mmol/L)	138.0 ± 3.2	137.1 ± 4.4	0.080	
Potassium (mmol/L)	4.1 ± 0.4	4.3 ± 0.5	0.009 *	Δ −0.15 (95% CI −0.27 to −0.04)
Chloride (mmol/L)	100.0 ± 5.0	100.5 ± 5.9	0.483	
Calcium (mmol/L)	2.2 [2.1–2.3]	2.0 [1.8–2.2]	<0.001 *	
Bicarbonate (mmol/L)	23.8 ± 3.2	22.7 ± 3.4	0.014 *	Δ 1.10 (95% CI 0.23 to 1.96)
Hematology/Inflammation & Coagulation				
White blood cells (10^9^/L)	10.8 [7.8–15.4]	13.2 [9.4–17.4]	0.023 *	
Neutrophils (10^9^/L)	6.5 [4.6–9.0]	8.6 [5.6–11.5]	0.005 *	
Lymphocytes (10^9^/L)	1.1 [0.8–1.7]	1.0 [0.7–1.5]	0.164	
Hematocrit (%)	41.1 ± 6.1	42.2 ± 6.3	0.151	
Platelets (10^9^/L)	214.6 ± 70.9	202.5 ± 77.8	0.224	
C-reactive protein (mg/L)	31.6 [6.4–149.1]	23.3 [6.9–338.3]	0.886	
Procalcitonin (ng/mL])	0.07 [0.01–0.55]	0.07 [0.01–0.55]	0.598	
Lactate dehydrogenase (U/L)	245.9 ± 102.9	270.7 ± 134.7	0.148	
Prothrombin time (s)	11.8 ± 2.1	12.4 ± 2.2	0.040 *	Δ −0.59 (95% CI −1.15 to −0.03)
Activated partial thromboplastin time (s)	30.1 ± 5.1	31.4 ± 5.6	0.060	
International normalized ratio	1.00 ± 0.18	1.04 ± 0.18	0.087	
D-dimer (mg/L FEU)	0.88 [0.24–2.85]	1.77 [0.42–5.62]	0.011 *	

SAP, severe acute pancreatitis; IQR, interquartile range; CI, confidence interval; Alb, albumin; ALP, alkaline phosphatase; ALT, alanine aminotransferase; AST, aspartate aminotransferase; GGT, gamma-glutamyl transferase; TBil, total bilirubin; DBil, direct bilirubin; BUN, blood urea nitrogen; CRP, C-reactive protein; PCT, procalcitonin; LDH, lactate dehydrogenase; PT, prothrombin time; aPTT, activated partial thromboplastin time; INR, international normalized ratio. * indicates statistical significance at *p* < 0.05.

**Table 3:** Performance of 36 Feature Selection–Model Combinations.

Feature Selection	Model	AUROC (95% CI)	F1	Precision	Recall	Log Loss	Brier
Recursive Feature Elimination (RF)	k-Nearest Neighbors	0.826 (0.686–0.965)	0.319	0.204	0.733	0.504	0.159
Elastic Net Selection	Logistic Regression (Elastic Net)	0.795 (0.661–0.929)	0.302	0.211	0.533	0.503	0.154
Elastic Net Selection	Support Vector Machine (RBF)	0.786 (0.637–0.936)	0.421	0.348	0.533	0.399	0.111
Boruta	Logistic Regression (Elastic Net)	0.782 (0.642–0.922)	0.320	0.229	0.533	0.521	0.162
Boruta	Extreme Gradient Boosting (XGBoost)	0.775 (0.628–0.921)	0.348	0.258	0.533	0.663	0.235
LASSO (L1) Selection	k-Nearest Neighbors	0.771 (0.616–0.927)	0.317	0.208	0.667	0.505	0.158
Recursive Feature Elimination (RF)	Random Forest (ranger)	0.769 (0.622–0.916)	0.444	0.381	0.533	0.284	0.077
Boruta	Random Forest (ranger)	0.759 (0.589–0.928)	0.485	0.444	0.533	0.316	0.085
Boruta	Support Vector Machine (RBF)	0.752 (0.596–0.909)	0.421	0.348	0.533	0.360	0.098
Recursive Feature Elimination (RF)	Support Vector Machine (RBF)	0.750 (0.593–0.907)	0.308	0.216	0.533	0.470	0.139
Elastic Net Selection	k-Nearest Neighbors	0.747 (0.583–0.911)	0.286	0.182	0.667	1.037	0.157
Elastic Net Selection	Extreme Gradient Boosting (XGBoost)	0.744 (0.582–0.905)	0.367	0.265	0.600	0.664	0.235
LASSO (L1) Selection	Extreme Gradient Boosting (XGBoost)	0.742 (0.581–0.904)	0.389	0.333	0.467	0.288	0.080
Elastic Net Selection	Random Forest (ranger)	0.739 (0.570–0.908)	0.471	0.421	0.533	0.330	0.090
Recursive Feature Elimination (RF)	Logistic Regression (Elastic Net)	0.739 (0.580–0.898)	0.356	0.267	0.533	0.584	0.196
Elastic Net Selection	Multivariate Adaptive Regression Splines	0.737 (0.594–0.881)	0.226	0.149	0.467	0.609	0.181
Univariate AUC filter	Support Vector Machine (RBF)	0.736 (0.570–0.902)	0.286	0.195	0.533	0.524	0.160
Minimum Redundancy–Maximum Relevance	Random Forest (ranger)	0.735 (0.572–0.898)	0.500	0.538	0.467	0.253	0.065
Recursive Feature Elimination (RF)	Multivariate Adaptive Regression Splines	0.729 (0.563–0.895)	0.277	0.180	0.600	0.536	0.162
LASSO (L1) Selection	Random Forest (ranger)	0.727 (0.556–0.897)	0.500	0.471	0.533	0.275	0.072
Recursive Feature Elimination (RF)	Extreme Gradient Boosting (XGBoost)	0.726 (0.548–0.904)	0.432	0.364	0.533	0.693	0.250
Boruta	Multivariate Adaptive Regression Splines	0.724 (0.567–0.881)	0.208	0.123	0.667	0.542	0.178
LASSO (L1) Selection	Support Vector Machine (RBF)	0.722 (0.555–0.889)	0.286	0.206	0.467	0.441	0.128
Univariate AUC filter	Logistic Regression (Elastic Net)	0.715 (0.539–0.890)	0.348	0.258	0.533	0.578	0.193
Minimum Redundancy–Maximum Relevance	Extreme Gradient Boosting (XGBoost)	0.713 (0.554–0.871)	0.387	0.375	0.400	0.276	0.074
Univariate AUC filter	Random Forest (ranger)	0.706 (0.528–0.883)	0.467	0.467	0.467	0.281	0.074
Boruta	k-Nearest Neighbors	0.689 (0.540–0.837)	0.200	0.120	0.600	1.041	0.214
Minimum Redundancy–Maximum Relevance	Logistic Regression (Elastic Net)	0.679 (0.481–0.878)	0.286	0.195	0.533	0.548	0.178
LASSO (L1) Selection	Logistic Regression (Elastic Net)	0.673 (0.489–0.857)	0.314	0.222	0.533	0.612	0.210
Minimum Redundancy–Maximum Relevance	Multivariate Adaptive Regression Splines	0.670 (0.511–0.828)	0.238	0.185	0.333	0.349	0.102
Minimum Redundancy–Maximum Relevance	Support Vector Machine (RBF)	0.666 (0.467–0.866)	0.291	0.200	0.533	0.525	0.163
Univariate AUC filter	Multivariate Adaptive Regression Splines	0.664 (0.516–0.813)	0.179	0.122	0.333	0.564	0.153
Minimum Redundancy–Maximum Relevance	k-Nearest Neighbors	0.664 (0.476–0.852)	0.207	0.125	0.600	1.355	0.214
Univariate AUC filter	Extreme Gradient Boosting (XGBoost)	0.659 (0.484–0.834)	0.364	0.333	0.400	0.322	0.076
LASSO (L1) Selection	Multivariate Adaptive Regression Splines	0.640 (0.474–0.806)	0.203	0.136	0.400	0.550	0.169
Univariate AUC filter	k-Nearest Neighbors	0.595 (0.403–0.787)	0.128	0.071	0.600	0.797	0.292

RF = Random Forest, RBF = Radial Basis Function, XGBoost = Extreme Gradient Boosting, LASSO = Least Absolute Shrinkage and Selection Operator.

## Data Availability

The data supporting the findings of this study are available upon reasonable request from the corresponding author. Due to privacy and ethical restrictions, the dataset cannot be publicly shared.

## Supplementary Materials

The following supporting information can be downloaded at: https://www.mdpi.com/article/10.3390/diagnostics15192473/s1, Table S1: Variables Selected by Different Feature Selection Methods.

## Abbreviations

The following abbreviations are used in this manuscript:

AP	Acute pancreatitis
APACHE II	Acute Physiology and Chronic Health Evaluation II
AUROC	Area under the receiver operating characteristic curve
BISAP	Bedside Index for Severity in Acute Pancreatitis
ED	Emergency department
GCS	Glasgow Coma Scale
ICU	Intensive care unit
kNN	k-nearest neighbors
LASSO	Least absolute shrinkage and selection operator
MARS	Multivariate adaptive regression splines
mRMR	Minimum redundancy–maximum relevance
RF	Random forest
RFE	Recursive feature elimination
RFE-RF	Recursive feature elimination using a random-forest estimator
ROC	Receiver operating characteristic
SAP	Severe acute pancreatitis
SHAP	Shapley additive explanations
SVM-RBF	Support vector machine with radial basis function kernel
XGBoost	Extreme gradient boosting
