# Supplementary material for: Integrating Feature Selection, Machine Learning, and SHAP Explainability to Predict Severe Acute Pancreatitis
Source: Diagnostics (Basel). 2025 Sep 27;15(19):2473. doi: 10.3390/diagnostics15192473 (PMC12523390; doi:10.3390/diagnostics15192473)
Supplement: Supplementary file 1 [file diagnostics-15-02473-s001.zip › diagnostics-3881549-supplementary.pdf]

Supplementary Table S1. Variables Selected by Different Feature Selection Methods

Method	n Variables	Selected Variables
Recursive Feature Elimination (RF)	8	alb, amyl, ca, creat, crp, gcs, lip, spo2
Boruta	15	alb, amyl, ast, bun, ca, cl, creat, gcs, ggt, hr, ldh, malign, rr, SI, spo2
Elastic Net Selection	8	alb, amyl, ca, creat, crp, gcs, lip, tbil
LASSO (L1) Selection	11	alb, amyl, bun, ca, creat, gcs, ggt, glucose_mgdl, malign, pct, pleural_eff
Minimum Redundancy–Maximum Relevance	25	alb, alt, amyl, ast, bun, ca, cad, copd, creat, crp, ddimer, dm, etiology, gcs, glucose_mgdl, ht, lip, male, malign, neut, pct, peripan_fl, pleural_eff, rr, spo2
Univariate AUC Filter	25	alb, alp, aptt, bun, ca, creat, dbil, ddimer, etiology, gcs, ggt, glucose_mgdl, hco3, hct, hr, k, ldh, malign, neut, peripan_fl, pleural_eff, pt, rr, SI, spo2

alb, albumin; amyl, amylase; alt, alanine aminotransferase; alp, alkaline phosphatase; aptt, activated partial thromboplastin time; ast, aspartate aminotransferase; bun, blood urea nitrogen; ca, calcium; cad, coronary artery disease; cl, chloride; copd, chronic obstructive pulmonary disease; creat, creatinine; crp, C-reactive protein; dbil, direct bilirubin; ddimer, D-dimer; dm, diabetes mellitus; etiology, pancreatitis etiology; gcs, Glasgow Coma Scale; ggt, gamma-glutamyl transferase; glucose_mgdl, serum glucose (mg/dL); hco3, bicarbonate; hct, hematocrit; hr, heart rate; ht, hypertension; k, potassium; ldh, lactate dehydrogenase; lip, lipase; malign, malignancy; male, sex (male); neut, neutrophil count; pct, procalcitonin; peripan_fl, peripancreatic fluid collection; pleural_eff, pleural effusion; pt, prothrombin time; rr, respiratory rate; sbp, systolic blood pressure; SI, shock index; spo2, oxygen saturation; tbil, total bilirubin.
